# Enhanced phenylpropanoid metabolism underlies resistance to *Fusarium oxysporum* f. sp. *vasinfectum* race 4 infection in the cotton cultivar Pima-S6 (*Gossypium barbadense* L.)

**DOI:** 10.3389/fgene.2023.1271200

**Published:** 2024-01-08

**Authors:** Jonathan Odilón Ojeda-Rivera, Mauricio Ulloa, Francisco G. Pérez-Zavala, Héctor-Rogelio Nájera-González, Philip A. Roberts, Lenin Yong-Villalobos, Himanshu Yadav, Ricardo A. Chávez Montes, Luis Herrera-Estrella, Damar Lopez-Arredondo

**Affiliations:** ^1^ Institute of Genomics for Crop Abiotic Stress Tolerance, Plant and Soil Science Department, Texas Tech University, Lubbock, TX, United States; ^2^ Plant Stress and Germplasm Development Research, U.S. Department of Agriculture-Agricultural Research Service, Plains Area, Cropping Systems Research Laboratory, Lubbock, TX, United States; ^3^ Department of Nematology, University of California, Riverside, CA, United States; ^4^ Unidad de Genomica Avanzada/Langebio, Centro de Investigacion y de Estudios Avanzados, Irapuato, Guanajuato, Mexico

**Keywords:** Pima cotton, *F. oxysporum* race 4-resistance, phenylpropanoid metabolism, Fusarium wilt, RNA-seq analysis

## Abstract

**Introduction:** *Fusarium oxysporum* f. sp. vasinfectum (FOV) race 4 (FOV4) is a highly pathogenic soil-borne fungus responsible for Fusarium wilt in cotton (*Gossypium spp.*) and represents a continuing threat to cotton production in the southwest states of the United States, including California, New Mexico, and Texas. Pima (*G. barbadense* L.) cotton, which is highly valued for its fiber quality, has been shown to be more susceptible to this pathogen than Upland (*G. hirsutum* L.) cotton. Still, some Pima cultivars present resistance to FOV4 infection.

**Methods:** To gain insights into the FOV4-resistance mechanism, we performed comparative transcriptional and metabolomic analyses between FOV4-susceptible and FOV4-resistant Pima cotton entries. FOV4-resistant Pima-S6 and FOV4-susceptible Pima S-7 and Pima 3-79 cotton plants were infected with FOV4 in the greenhouse, and the roots harvested 11 days post-infection for further analysis.

**Results:** We found that an enhanced root phenylpropanoid metabolism in the resistant Pima-S6 cultivar determines FOV4-resistance. Gene-ontology enrichment of phenylpropanoid biosynthesis and metabolism categories correlated with the accumulation of secondary metabolites in Pima-S6 roots. Specifically, we found esculetin, a coumarin, an inhibitor of Fusarium’s growth, accumulated in the roots of Pima-S6 even under non-infected conditions. Genes related to the phenylpropanoid biosynthesis and metabolism, including phenylalanine ammonia-lyase 2 (PAL2) and pleiotropic drug resistance 12 (PDR12) transporter, were found to be upregulated in Pima-S6 roots.

**Discussion:** Our results highlight an essential role for the phenylpropanoid synthesis pathway in FOV4 resistance in Pima-S6 cotton. These genes represent attractive research prospects for FOV4-disease resistance and breeding approaches of other cotton cultivars of economic relevance.

## 1 Introduction

*Fusarium oxysporum* f. sp*. vasinfectum* (FOV) is a pathogenic soil-borne fungus responsible for Fusarium wilt in cotton (*Gossypium* spp.). For over a decade, the FOV race 4 (FOV4) strain has adversely impacted cotton production areas in the U.S., causing plant wilt and death (Ulloa et al., 2006; Hutmacher et al., 2013; Ulloa et al., 2013; Ulloa et al., 2016a; Ulloa et al., 2020). Upland (*G. hirsutum* L.) and Pima (*G. barbadense* L.) cotton account for around 95.5% and 4.5%, respectively, of total fiber production in the U.S., and Pima cotton represents a valuable genetic source of essential traits like fiber quality and disease resistance (Ulloa et al., 2013; Zhang et al., 2014; Ulloa et al., 2022a; Chávez Montes et al., 2023). Pima fiber is highly valued in the cotton industry because its extra-long, strong, and fine fibers are best suited to produce high-quality fabrics. Previous studies have shown that Pima cotton cultivars are more susceptible to FOV4 than Upland, and Pima S-7 and Pima 3-79 have been used as susceptible checks in many of these studies (Ulloa et al., 2006; Ulloa et al., 2013; Ulloa et al., 2020; Zhang et al., 2022). Fortunately, some Pima cultivars, such as Pima S-6, identified in 2004, present resistance to FOV4 infection (Feaster and Turcotte, 1984; Ulloa et al., 2006). Pima S-6 has been subjected to several cycles of evaluation and selection under naturally FOV4-infested fields to increase its uniformity and resistance level. This new source, renamed Pima-S6, has been incorporated into cotton breeding programs to combat the highly pathogenic FOV4 in the U.S. (Ulloa et al., 2016b; Ulloa et al., 2022a; Ulloa et al., 2022b).

Previous QTL and GWAS studies in interspecific mapping populations (Upland × Pima) and Upland cotton have provided insights into the genetic basis of FOV4 resistance in this crop (Ulloa et al., 2013; Ulloa et al., 2016a; Wang et al., 2018; Zhang et al., 2022; Zhu et al., 2022). Some studies have linked specific *G. barbadense* chromosomes to FOV4 resistance (Ulloa et al., 2013; Ulloa et al., 2016a). However, specific genes underlying FOV4 resistance in Pima cotton have yet to be found and characterized. While these studies establish that FOV4 resistance is present in specific cultivars of Pima cotton, the underlying functional mechanisms for disease resistance are unknown. Because Pima-S6 is resistant to FOV4 and Pima S-7 and Pima 3-79 are not, these genotypes are suitable models to perform transcriptional profiling and determine the differences in gene expression in response to FOV4 infection.

In this work, we performed comparative transcriptional and metabolomic profiling of FOV4-resistant Pima-S6 and FOV4-susceptible Pima S-7 and Pima 3-79 cotton accessions to gain insights into the mechanisms underlying FOV4 resistance. Transcriptomic profiling enabled the identification of candidate genes potentially involved in the resistance mechanism of FOV4, protecting the Pima-S6 cotton cultivar from this pathogenic fungus. Our results highlight an essential role for the phenylpropanoid synthesis pathway in the resistance to FOV4 in Pima-S6, which represents a source of key metabolites for plant development and environmental adaptation (Fraser and Chapple, 2011).

## 2 Materials and methods

### 2.1 Source of plant material

*Gossypium barbadense* inbred lines Pima S-7 (PI 560140) and Pima 3-79 susceptible to *Fusarium oxysporum* f. sp. *vasinfectum* (FOV) race 4 (FOV4), and Pima-S6 resistant to FOV4, were used in this study. Pima entries are public lines released previously (Feaster and Turcotte, 1984; Turcotte et al., 1992; Kim et al., 2005). Pima-S6, specifically, was derived from the original source Pima S-6 after being identified as an important source of resistance traits to FOV4 (Ulloa et al., 2006; Ulloa et al., 2020; Ulloa et al., 2022a). The new source was named Pima-S6, as explained in (Chávez Montes et al., 2023).

### 2.2 *Fusarium* wilt race 4 (FOV4) infection of Pima roots

The three Pima entries were used to prepare cDNA libraries and RNA-seq analyses. The FOV4 inoculum was produced in potato dextrose liquid medium supplemented with 3 mM streptomycin to prevent contamination and incubated at 28°C and constant shaking at 100 rpm during 3–4 days. We utilized plugs (2–3 mm^2^) of FOV4 fresh-grown onto potato dextrose agar (PDA, streptomycin), as the starting material. Finally, a conidial suspension (1 × 10^6^ conidia/mL) was generated by filtering the culture through cheesecloth.

The infection of the roots in the greenhouse was performed according to the root-cut dip method as previously described (Ulloa et al., 2013). Briefly, uprooted one-week-old cotton seedlings were dipped in the FOV4 conidia suspension and transplanted into sterilized U.C. mix #2 substrate after 3 min. Five treated plants were planted per pot and incubated in the greenhouse at 24°C ± 2°C. None inoculated plants treated with water only were used as controls. Four replicates for all treatments were used following a randomized complete block design. At 11 days after inoculation (dai), the plant and soil ball were removed from the pots, and the soil was removed gently from the roots by washing under continuous water flow. The washed roots were placed immediately in 50 mL screw-top plastic vials, liquid nitrogen was added, and the vials were placed in a −80°C freezer with the tops off to allow vaporization of the liquid nitrogen. Then the tops were screwed on, and the frozen roots were stored at −80°C until RNA extraction.

### 2.3 RNA-seq data analysis

We used a similar RNA-seq data processing pipeline as that reported in (Ojeda-Rivera et al., 2022). Briefly, quality control of Illumina reads was performed using FastQC (version 0.11.9; https://www.bioinformatics.babraham.ac.uk/projects/fastqc/). Adapter sequences were removed from libraries with Trimmomatic (version 0.39) (Bolger et al., 2014). We then quantified gene expression using kallisto (version 0.46.1) (Bray et al., 2016). Pima cotton libraries were aligned to our assembly of the Pima-S6 *G. barbadense* genome (Chávez Montes et al., 2023). edgeR was used to perform differential expression analysis (Robinson et al., 2010), the tximport package (version 3.130) (Soneson et al., 2015) was used to import estimated counts from kallisto to edgeR. We applied a base 2 logarithm of fold change (logFC) threshold (−1>logFC>1) and a false discovery (FDR) threshold (FDR<0.05) to detect differentially expressed genes. We used a similar pipeline for GO analysis to that reported in (Ojeda-Rivera et al., 2022). Briefly, annotations for the *G. barbadense* Pima-S6 genome (Chávez Montes et al., 2023) were assigned using the GO annotations of the blastp reciprocal best hits from multiple databases as reference. Databases included Araport11 (Cheng et al., 2017), UniProt (Apweiler et al., 2004), Interproscan (v5.48.83) (Blum et al., 2021), Swiss-Prot proteins from multiple plant species (Wang et al., 2021), and GO annotations from the PANNZER2 (Törönen et al., 2018) functional annotation webserver with a PPV value of at least 0.5. We combined analyses into a non-redundant gtf file and used that file for GO enrichment analyses using the clusterProfiler package for R (Wu et al., 2021). The datasets generated and analyzed for this study can be found in the National Center for Biotechnology Information (NCBI) Gene Expression Omnibus (GEO) database repository under accession number GSE233711.

### 2.4 Plant culture for metabolomic analysis

For non-targeted metabolomics analysis, non-infected roots of the three cotton entries, Pima-S6, Pima 3-79, and Pima S-7 were utilized. Cotton seedlings were grown vertically in Petri dishes in a Conviron growth chamber set at 28°C, 150 µM/m^2^s light intensity, 16/8 h light/dark cycle, and 45% relative humidity. Culture media was 0.25 X MS with slight modifications (pH 6, 5 mM CaCl_2_, and 1 mM MgCl_2_) and 0.35% Phytagel (Sigma-Aldrich). Root samples for metabolomic analysis were harvested 8 days after germination. Five biological replicates were performed (*n* = 5).

### 2.5 Metabolomic analysis

Non-targeted metabolomic analysis and quantitative analysis of esculetin were performed in non-infected cotton root samples. Non-targeted metabolomics analysis was conducted using a reverse phase chromatography method and positive electrospray ionization mode mass spectrometry. Freeze-dried samples of cotton roots were powdered and extracted with a mixture of methanol-water (60:40 v/v) with 0.1% formic acid. The supernatant of this extraction was further concentrated in a vacuum centrifuge (Labconco, United States) and filtered before chromatographic analysis. Chromatographic separation was performed in a Vanquish Flex UHPLC system (Thermo Scientific, United States) using an Acclaim VANQUISH column (150 × 2.1 mm, with 2.2 µm) and a solvent gradient method starting 5% phase B for 4 min, then increasing to 100% B from 4–17 min, and finally maintaining 100% B for 4 min prior to re-equilibration time of 4 min. Phases consisted of Water (A) and Methanol (B) supplemented with 0.1% formic acid. Detection was carried out in an Orbitrap Exploris 240 (Thermo Scientific, United States) with electrospray ionization source in positive mode scanning from 70–800 Da with a resolution of 120,000 and Data Dependent Acquisition (DDA) of MS/MS with a cycle time of 3 s. Raw files were imported into Compound Discoverer 3.3 (Thermo Fisher Scientific) and processed with an untargeted metabolomics workflow. Chromatograms were aligned, compounds were detected and grouped with a 5 ppm tolerance window. Detected metabolites were identified using MZCloud MS/MS database with a similitude factor of at least 50%. Exact masses were also searched against BioCyc and KEGG databases (Wu et al., 2021), and the final lists were curated manually. Pathway enrichment analysis was done in the MetaboAnalyst online platform (Pang et al., 2022), using the exact m/z values of detected compounds for the MS Peak-to-Pathway enrichment (Li et al., 2013), retention times, and statistical values from Compound Discoverer analysis. The KEGG pathway analysis presented in Figure 4D was generated by graphing the results of the Metaboanalyst platform (Pang et al., 2022), which uses the KEGG database as a reference (Kanehisa et al., 2023), in the ComplexHeatmap R package (Bolger et al., 2014). Figure 5B, an illustration of the phenylpropanoid pathway, was generated from our gene-by-gene analysis of Arabidopsis orthologs in cotton.

Quantitative analysis of esculetin was done using an external standard methodology. High-purity esculetin (>98%) was acquired from Cayman Chemical (item:19286) and injected into the same UHPLC-MS system described before using a modified method. The separation column was a Waters BEH C18 (2.1 × 150 mm, 2.5 µm) with water and methanol added with 0.1% formic acid as mobile phases. The retention time for esculetin was 6.36 min, when methanol concentration in the chromatographic method was 50%. Detection in the Orbitrap MS was carried out in negative mode, using H-ESI. A full MS scan from m/z = 100–300 with a resolution of 120,000 preceded a targeted MS/MS using an intensity threshold filter of 5 × 103 and targeted mass for esculetin [M-H]- = 177.0193 ± 10 ppm. Collision energy for the MS/MS fragmentation was normalized to 30% and resolution to 15,000. A calibration curve from 10–10000 ng/mL was injected in duplicate (r.s.d. < 10%) with R2 = 0.9978 and used to quantify samples (*n* = 3) fragmentation pattern of the standard.

### 2.6 Real-time PCR

Total RNA was isolated from the root tissue of non-infected cotton plants, as described in previous sections. 100 ng of total RNA was used to carry out the reverse transcription reaction with SuperScript III reverse transcriptase (Invitrogen), following the manufacturer’s protocol. The RT-qPCR was performed in a MIC thermocycler (Magnetic Induction Cycler from Biomolecular Systems) using the reagent SensiFast TM SYBR No-ROX Kit from Bioline (BIO-11060). The PCR conditions were 95°C for 2 min, followed by 40 cycles at 95°C for 5 s, 65°C for 10 s, and 72°C for 20 s. The relative expression of each gene was calculated relative to the mean of the Cq of POLYUBIQUITIN 14 (UBQ14) gene for each sample. UBQ14 qPCR was made using previously reported primers (Artico et al., 2010). Primers were designed for PHENYLALANINE AMMONIA-LYASE 2 (PAL2) and PLEIOTROPIC DRUG RESISTANCE 12 (PDR12); the forward primer 5′-CAT​TGT​GAG​CCA​AGT​TGC​TAA​G-3′ and reverse primer 5′-GTC​CAC​GGC​TTT​GAG​TAA​GT-3′ for PAL2 gene and the forward primer 5′-GGA​GAA​TGG​TGA​GGC​ATT​TAG​A-3′ and reverse primer 5′-CAC​GAG​CAG​ACA​TGG​AGA​AA-3′ for PDR12) gene. To calculate the FC values, the Pfaffl formula, which corrects the FC accounting for differences in primer PCR efficiencies, was used (Pfaffl, 2001).

## 3 Results

### 3.1 FOV4-resistant Pima-S6 presents a specific transcriptional response to FOV4 infection

**FIGURE 1:**
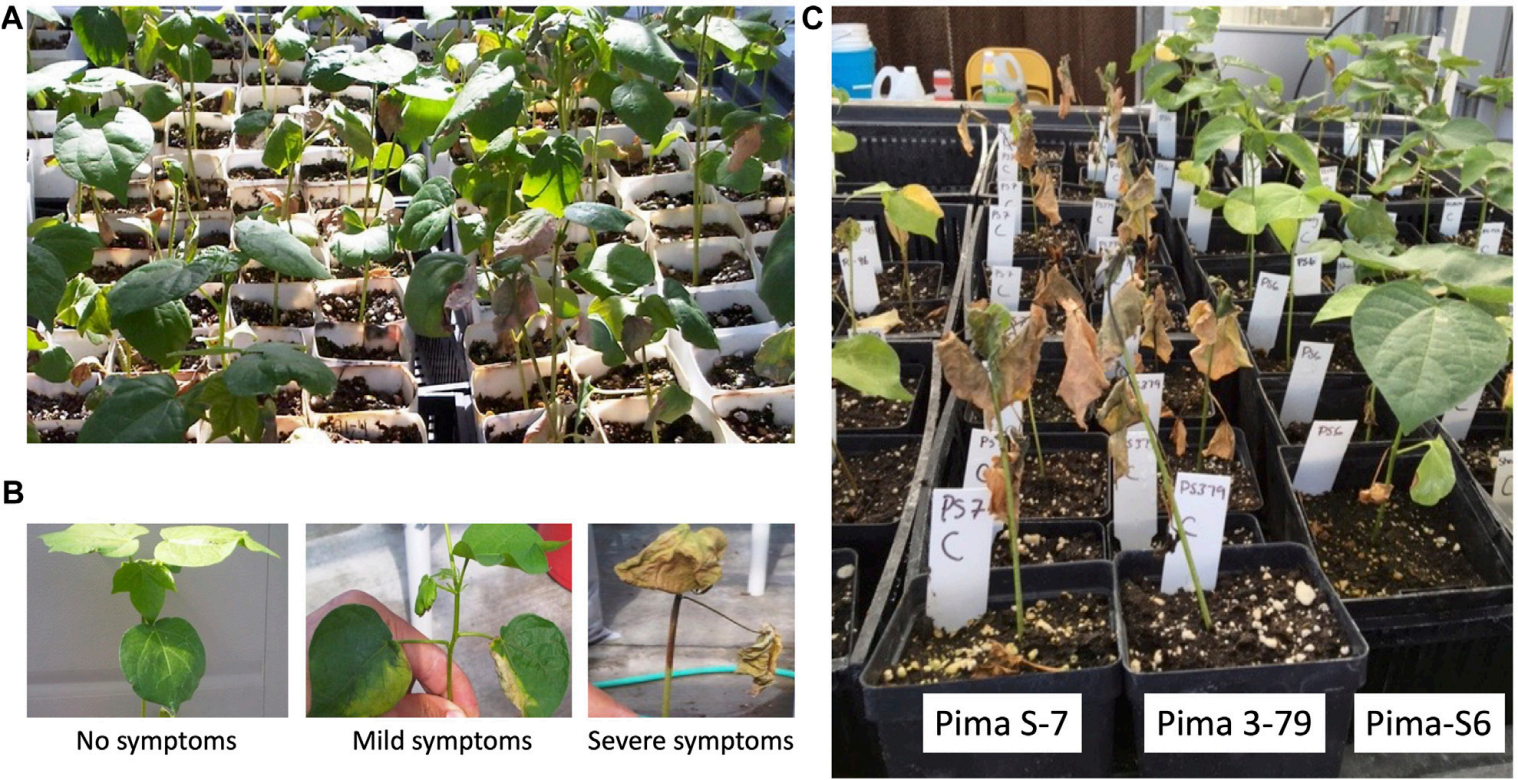
Pima-S6 shows resistance to FOV4. Representative photographs of greenhouse evaluations using artificial inoculation of Fusarium wilt (*Fusarium oxysporum* f. sp. *vasinfectum*) race 4 (FOV4) infection (10^−6^) in the three cotton entries: susceptible Pima S-7 and Pima 3-79, and resistant Pima-S6, 18 days after inoculation. **(A)** replicates randomly distributed in the greenhouse bench; **(B)** symptoms manifested by treated plants; **(C)** replicates of the three cotton entries grouped to take a photograph.

To characterize the transcriptional response to Fusarium wilt disease caused by FOV4 in Pima cotton, we performed RNA-seq analysis of plant roots grown under control and FOV4-infected conditions. Three Pima cotton cultivars were studied: one FOV4-resistant cultivar, Pima-S6, and two FOV4-susceptible cultivars, Pima S-7 and Pima 3-79 (see Materials and Methods). Plants of the three cotton entries were visually inspected after FOV4 infection throughout the duration of the experiment, looking for disease symptoms. Infected susceptible plants exhibited severe leaf wilt, chlorosis, and leaf abscission, leading to plant death, as reported previously (Ulloa et al., 2013). Resistant plants showed normal growth and minimal symptoms, and survived FOV4-infection. Representative photographs of infection experiments are presented in Figure 1. Based on our observations on disease severity, it was determined that 11 day was appropriate for tissue harvesting and conducting RNA-seq studies. Otherwise, advanced disease symptoms would prevent high-quality total RNA extraction. To this end, three biological replicates per treatment per cotton entry were used to generate 18 RNA-seq libraries. Alignment statistics and library names are listed in Supplementary Table S1.

**FIGURE 2:**
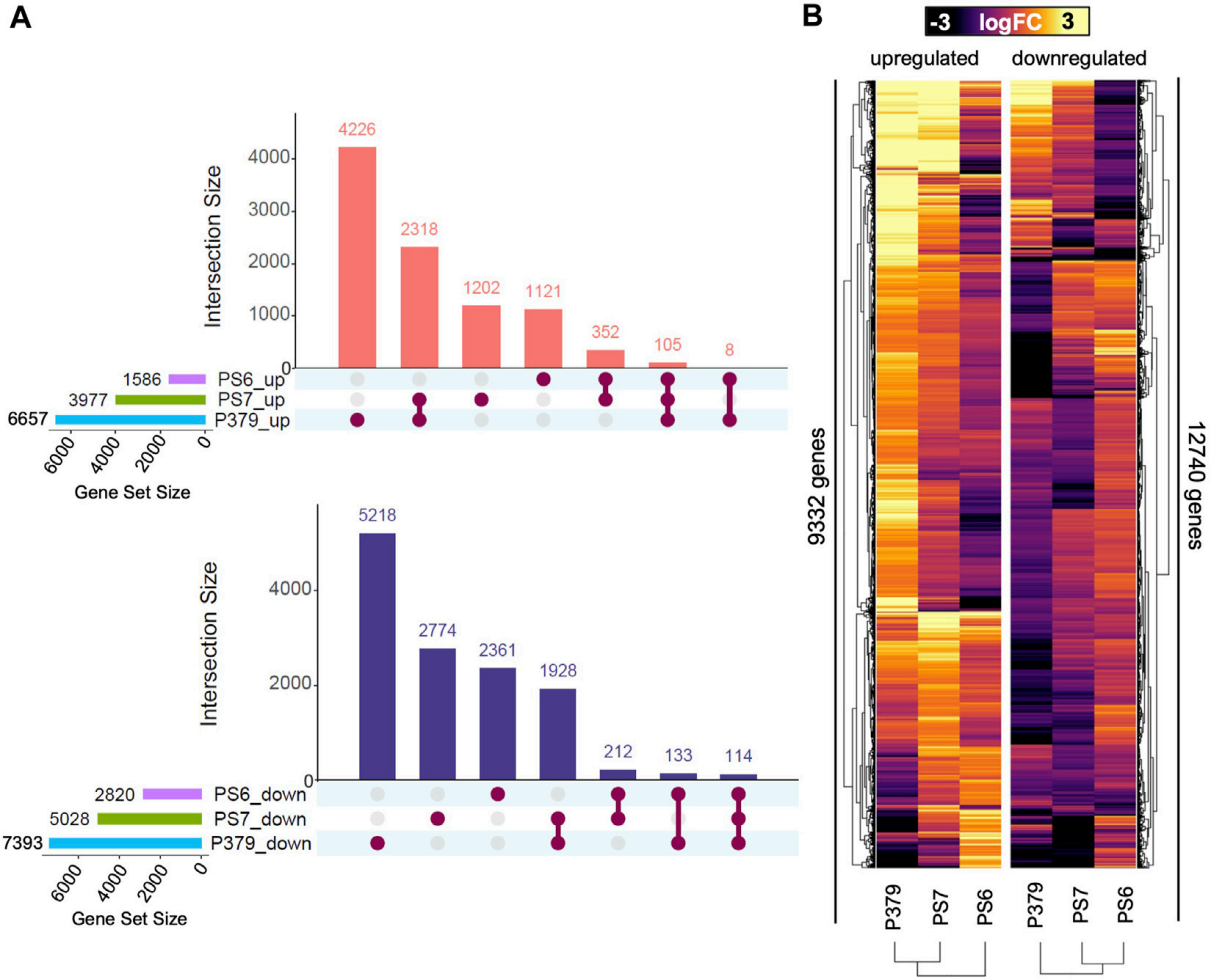
Pima-S6 presents a specific and compact transcriptomic response to FOV4 infection compared to Pima S-7 and Pima 3-79. **(A)** Gene set analysis of shared and unique differentially expressed genes in response to FOV4 treatment in Pima cotton cultivars, Pima-S6 (PS6), Pima 3-79 (P379), and Pima S-7 (PS7). Connected dots represent common gene sets. Upper panel: upregulated genes; lower panel: downregulated genes. **(B)** Heatmap of the expression level of differentially upregulated (left section) or downregulated (right section) genes in logarithm of fold change (logFC) units, with statistical significance in at least one of the three genotypes tested.

To get insights into the molecular mechanisms underlying FOV4 resistance, we first performed a comparative analysis of the transcriptional response of all FOV4-susceptible (Pima S-7 and Pima 3-79) and resistant (Pima-S6) cultivars (Figure 2). We performed sample relationship analysis to determine whether the samples clustered by the tested genotypes and treatments. RNA-seq libraries were observed to cluster together in response to both genotype and treatment conditions (Supplementary Figure S1). Susceptible Pima 3-79 and Pima S-7 cultivars present similar expression profiles in response to FOV4 infection as they cluster closely in the sample relationship analyses, while Pima-S6 sample libraries cluster more closely and separated from those of Pima 3-79 and Pima S-7 in response to FOV4 treatments (Supplementary Figure S1). This result indicates a differential response to FOV4 infection in Pima-S6 compared to Pima S-7 and Pima 3-79.

To further analyze the differences in response to FOV4 infection among the tested cultivars, we performed a comparative transcriptomic response analysis to determine which genes are commonly regulated in response to FOV4 treatment (Figure 2A). We determined that 6,657 and 7,393 genes were upregulated and downregulated in Pima 3-79 in response to FOV4 treatment (Figure 2A) and that up to 63% and 70% of these gene sets are specific to this cultivar. In the case of Pima S-7, we detected the upregulation and downregulation of 3,977 and 5,028 genes, respectively, in response to FOV4 treatment (Figure 2A), of which 30% and 55% were specific to this cultivar. Lastly, in the case of Pima-S6, we detected that 1,586 and 2,820 genes were upregulated and downregulated, respectively, in response to FOV4 treatment (Figure 2A), of which 70% and 83% of these gene sets are specific to Pima-S6. Genes commonly upregulated and downregulated in susceptible cultivars Pima S-7 and Pima 3-79 are 2,318 and 1,928, respectively, which correspond to a ∼47% of the Pima S-7 transcriptional response to FOV4 (∼30% of the Pima 3-79 response), indicating that a relatively large number of genes of the response to FOV4 is commonly regulated in susceptible cultivars. In contrast, Pima-S6 and Pima S-7 had only 352 upregulated and 212 downregulated genes in common, corresponding to ∼6% and ∼12% of the FOV-4 responsive genes in Pima S-7 and Pima-S6, respectively. Considering the three genotypes, 105 genes were upregulated and 114 downregulated in response to FOV4; this number equals less than 4% of the global transcriptional response for all the genotypes. The transcriptomic landscape of the response to FOV4 (Figure 2B) agrees with the observed differences in gene expression between susceptible and resistant cultivars. It also indicates differences in the magnitude of the fold change in gene expression. Overall, our data showed that susceptible genotypes share a more significant portion of their transcriptional response to FOV4 among themselves than with Pima-S6, and that the transcriptional response of the resistant cultivar is specific and different from that of the susceptible cultivars.

### 3.2 Gene Ontology (GO)-enriched categories in Pima-S6 in response to FOV4 relate to phenylpropanoid metabolism

**FIGURE 3:**
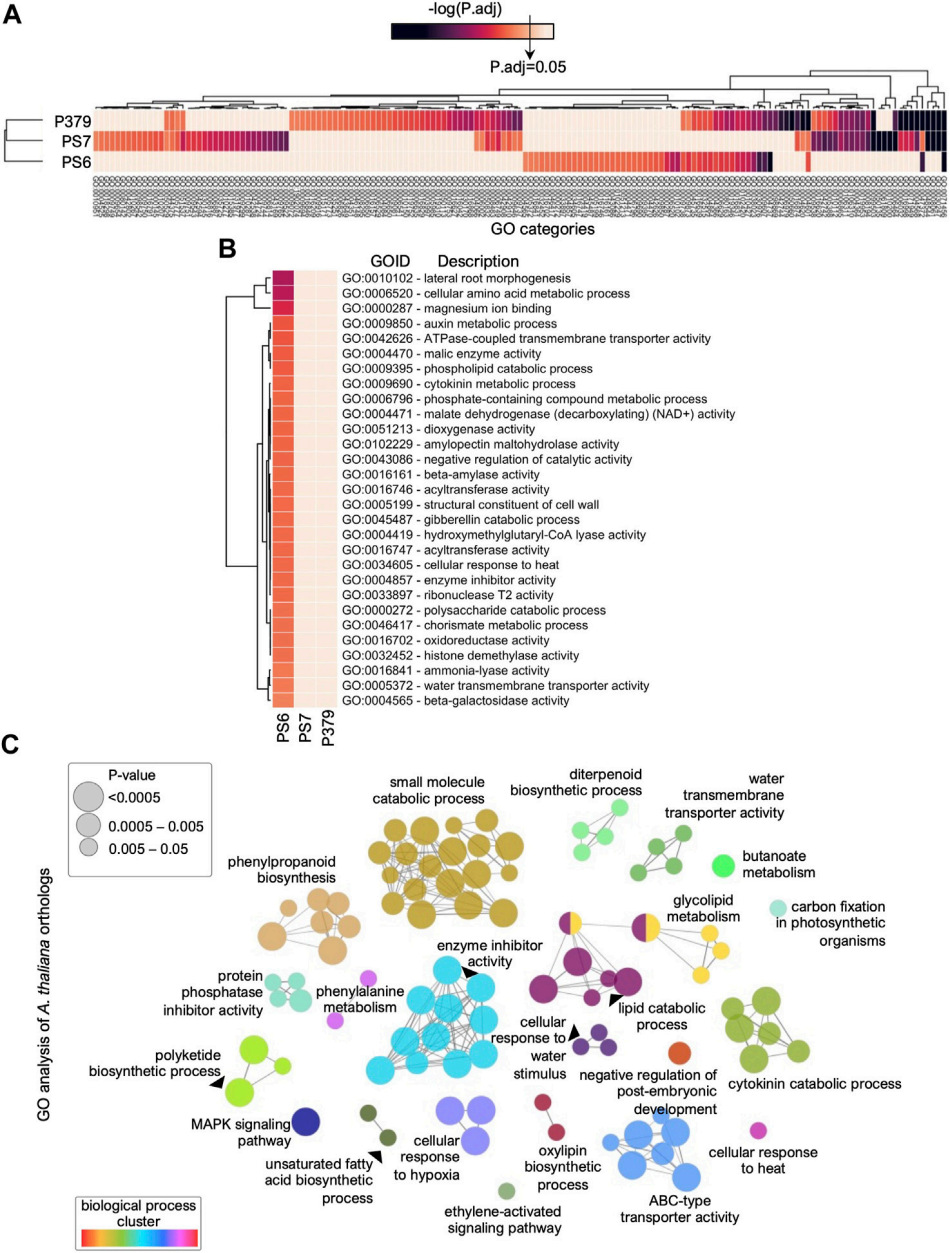
Differential analysis of Gene Ontology (GO) enriched categories in Pima cotton cultivars indicates secondary metabolism-related processes are activated in Pima-S6 in response to FOV4 infection. **(A)** Heatmap representing GO categories found enriched in at least one of the cultivars tested, Pima-S6 (PS6), Pima 3-79 (P379), and Pima S-7 (PS7). The statistical significance key, -log(P.adj), indicates the threshold for enrichment. **(B)** Enriched GO categories specific to the Pima-S6 resistant genotype in response to FOV4 treatment. **(C)** GO Enrichment analysis of the Arabidopsis orthologs from **(B)**. Each bubble represents a GO category, and connections represent shared genes between categories. Categories are clustered according to the biological process. A full list of GO categories and descriptions from **(A–C)** is included in Supplementary Dataset S1.

To get biological insights into the FOV4-resistance mechanism(s) that are transcriptionally activated in Pima-S6 in response to FOV4 treatment, we performed differential Gene Ontology (GO) enrichment analysis (Figure 3). The complete list of GO-enriched categories, *p*-values, and gene identifiers is included in Supplementary Dataset S1. First, we determined the GO categories enriched in the gene sets that are transcriptionally activated in Pima 3-79, Pima-S6, and Pima S-7, respectively, in response to FOV4 treatment (Figure 3A). We then focused our analysis on categories significantly enriched only in Pima-S6, the FOV4-resistant genotype (Figure 3B). The identified GO categories presented in Figure 3B are related to a variety of biological processes including transmembrane transport (GO:0042626, GO:0005372), glycoside hydrolysis (GO:0016161, GO:0102229, GO:0000272, GO:0004565), metabolism of small molecules and amino acids (GO:0006520, GO:0004471), phospholipid metabolism (GO:0009395), phytohormone metabolism (GO:0010102, GO:0009850, GO:0009690, GO:0051213) and phenylpropanoid metabolism (GO:0046417, GO:0016841). Because the Arabidopsis genome is more functionally characterized than the cotton genome, we searched for Arabidopsis orthologs of the genes included in these GO categories to get more insights into their possible roles in FOV4 resistance (Figure 3C, Supplementary Dataset S1). GO enrichment and KEGG pathway analysis (Kanehisa et al., 2023) of Arabidopsis ortholog genes (Figure 3C) resulted in multiple categories clustered to similar biological processes as the categories from Figure 3B. We found clusters of GO categories that include the metabolism of small molecules, phenylpropanoid biosynthesis and metabolism, ABC-type transporter activity, lipid metabolism, regulation of enzymatic activity, diterpenoid biosynthetic processes, and phytohormone signaling-related processes.

The phenylpropanoid pathway in plants is the starting point for other metabolic pathways that produce critical secondary metabolites, including flavonoids, coumarins, and lignans, which have roles in plant resistance to pathogens (Fraser and Chapple, 2011). Gene-by-gene analysis of Arabidopsis orthologs (Supplementary Dataset S1) allowed the identification of genes that encode key enzymes involved in the phenylpropanoid pathway, including PHENYLALANINE AMMONIA LYASE 1 and 2 (PAL1,2; PimaS6.A09G041328.1, PimaS6.A11G018686.1, PimaS6.D11G014299.1), which catalyze the initial step of the phenylpropanoid pathway (Huang et al., 2010), and CHORISMATE MUTASE 2 (CM2; PimaS6.A06G072050.1, PimaS6.D06G069084.1) which catalyzes the first committed step in the synthesis of Phenylalanine (Fraser and Chapple, 2011). We also found an ortholog of CHALCONE SYNTHASE (CHS; PimaS6.D05G007786.1, PimaS6.A08G034631.1, PimaS6.A12G026213.1, PimaS6.D12G022648.1) that encodes the enzyme that catalyzes the initial step in the synthesis of flavonoids (Dao et al., 2011). Root exudation of secondary metabolism compounds through ABC-type transporters has also been shown to play a role in plant-microbe interactions and resistance to fungal pathogens (Chaparro et al., 2013; Fourcroy et al., 2014; He et al., 2019). Concomitantly, we found multiple cotton genes encoding ABC-type transporters orthologs to multiple Arabidopsis ABC-type transporters. Some of these transporters have been reported to play roles in fungal pathogen resistance, like PLEIOTROPIC DRUG RESISTANCE 12 (PDR12), by facilitating root exudation of secondary metabolites (He et al., 2019). The differential transcriptional response of Pima-S6 compared with susceptible genotypes Pima S-7 and Pima 3-79 indicates that secondary metabolism might play a key role in the FOV4 resistance mechanism. Interestingly, we found that genes in GO categories related to phenylpropanoid regulation and metabolism (GO:0009698, GO:0009715), have a higher basal expression in Pima-S6, the FOV4-resistant cotton, compared to the susceptible varieties, which is enhanced upon FOV4 infection (Supplementary Figure S2). This suggests that the enhanced basal expression of these genes might contribute to the FOV4 resistance phenotype.

### 3.3 Non-targeted metabolomics analysis reveals Pima-S6 cotton has a robust root metabolome that is key to FOV4-resistance

**FIGURE 4:**
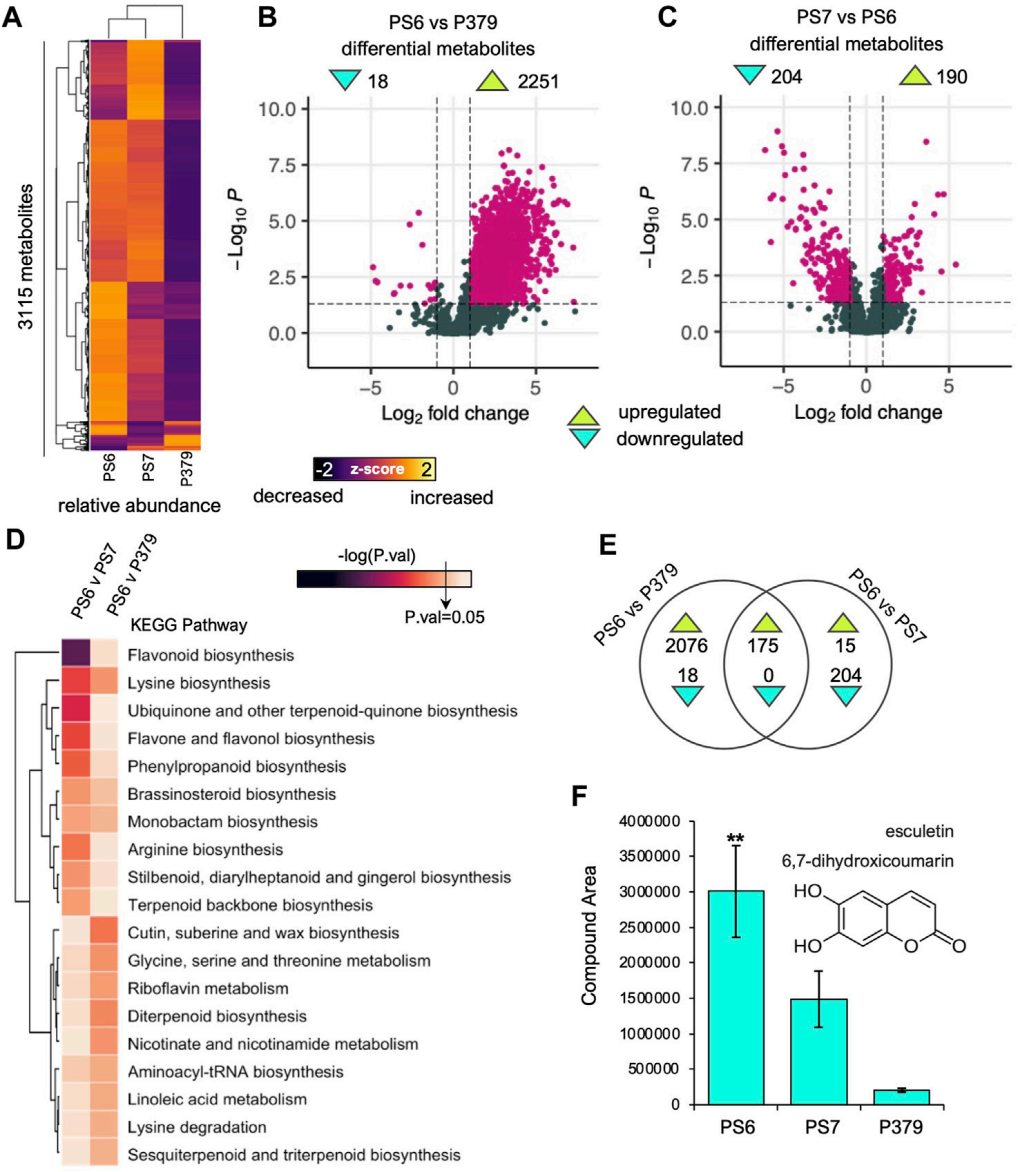
Mass spectrometry non-targeted metabolomic analysis of Pima-S6, Pima S-7, and Pima 3-79 corroborates differential enrichment of secondary metabolism-related compounds derived from the phenylpropanoid pathway in Pima-S6. **(A)** Heatmap of the normalized relative abundance (z-score) of the 3115 compounds identified using MS metabolomic profiling. **(B, C)** Volcano plots of the differential compounds found in the pairwise comparisons of Pima-S6 (PS6) vs. Pima 3-79 (P379) **(B)** and Pima-S6 vs. PS-7 (PS7) **(C)**. The threshold for a differential compound is (−1<logFC>1; *p*-value<0.05). **(D)** MS Peak-to-Pathway metabolite analysis. The key, -log(P.val), indicates the threshold for statistically significant enrichment of a KEGG metabolic pathway. **(E)** Venn analysis of differentially accumulated metabolites in the pairwise comparisons of PS6 vs. P379 and PS6 vs. PS7. **(F)** Relative abundance, in compound area units, of the coumarin esculetin in the three genotypes tested. Asterisks indicate that the level of this compound is significantly higher with statistical significance (P.val < 0.05).

To further explore the differences in secondary metabolism between the resistant and the susceptible genotypes, we performed mass spectrometry-based non-targeted metabolomics analysis in the roots of non-infected Pima-S6, Pima S-7, and Pima 3-79 seedlings (Figure 4). We detected 3,115 compounds in the roots of the three studied genotypes (Figure 4A). Cluster profiling of the normalized relative abundance of the identified compounds indicates that Pima-S6 and Pima S-7 metabolomes are more closely related to each other than to Pima 3-79 (Figure 4A). We performed pairwise comparisons to determine statistically significant differences in relative compound concentration of these 3115 metabolites between FOV4-resistant Pima-S6 and the susceptible genotypes, namely, Pima-S6 vs. Pima S-7 and Pima-S6 vs. Pima 3-79 (Figures 4B, C). We found 2,251 metabolites with higher levels and 18 with lower levels in Pima-S6 relative to Pima 3-79, indicating that Pima-S6 has a more diverse root metabolome than Pima 3-79 (Figure 4B). The Pima-S6 vs. Pima S-7 comparison showed that 204 metabolites have higher and 190 lower levels in Pima-S6 (Figure 4C), which agrees with the clustering profiling analysis (Figure 4A).

To get functional insights into the metabolomic differences between Pima-S6 and FOV4-susceptible genotypes (Pima-S6 vs. Pima S-7 and Pima-S6 vs. Pima 3-79), we performed a functional analysis using MS Peak-to-Pathway analysis (Li et al., 2013; Pang et al., 2022), which harnesses metabolite changes at pathway level to calculate enrichment and combines it with metabolite set enrichment analysis to determine an overall pathway enrichment *p*-value (Figure 4D; Supplementary Dataset S1). In the case of the pairwise comparison between Pima-S6 and Pima 3-79, it was determined that the “cutin, suberin, and wax biosynthesis” pathway was significantly more enriched in Pima-S6 than in Pima 3-79 (Figure 4D). Along with lignin, cutin, suberin, and wax are secondary cell wall components that, amidst other functions, provide protection against pathogen attack and interact metabolically with the phenylpropanoid synthesis pathway (Miedes et al., 2014; Andersen et al., 2021). In the case of the pairwise comparison between Pima-S6 and Pima S-7, six pathways were found significantly more enriched in Pima-S6 than in Pima S-7, including “flavonoid biosynthesis”, “lysine biosynthesis”, “ubiquinone and another terpenoid-quinone biosynthesis”, “flavone and flavonol biosynthesis” and “phenylpropanoid biosynthesis” (Figure 4D). All these categories, except for lysine biosynthesis, are closely related to secondary metabolism, specifically the phenylpropanoid pathway.

We rationalized that the set of metabolites key to FOV4-resistance in Pima-S6 must have enhanced levels compared to susceptible genotypes. To test this hypothesis, we performed Venn diagram analysis to determine more abundant metabolites in Pima-S6 compared to FOV4-susceptible Pima 3-79 and Pima S-7 genotypes (Figure 4E). We identified 175 compounds more abundant in Pima-S6 than in the two susceptible genotypes (Supplementary Dataset S1, spreadsheet 9.1). Their identity was determined by matching their spectra to known databases using the Compound Discoverer software (Thermo Scientific). We found 57 hits to known molecules (∼32%) among the Pima-S6-enhanced metabolites (Supplementary Dataset S1, spreadsheet 9.2). Inspection of the list revealed multiple molecules related to secondary metabolism, specifically phenylpropanoid metabolism, as suggested by the presence of a phenyl group in their structure (Supplementary Dataset S1), which agrees with the pathways presented in Figure 4D. Interestingly, we found enhanced levels of the coumarin esculetin in the roots of Pima-S6 (Figure 4F). Root coumarin exudation has been recently shown to inhibit *Fusarium oxysporum* f. sp*. raphani* growth *in vitro* and promote the establishment of plant-beneficial microorganism communities in the rhizosphere (Stringlis et al., 2018). A quantitative determination was performed using an external high-purity standard to corroborate the metabolite esculetin’s identity. UHPLC-MS analysis suggests that the fragmentation pattern of the esculetin standard and our samples matches and is one of the most abundant in Pima-S6 (Figure 4F; Supplementary Figure S3).

## 4 Discussion

Breeding programs have successfully developed FOV4-resistant cotton germplasm (Ulloa et al., 2016b; Ulloa et al., 2022a; Ulloa et al., 2022b). However, little is known about the molecular mechanisms underlying FOV4 resistance in this crop. To address this question, we harnessed our recent assembly of the Pima-S6 genome (Chávez Montes et al., 2023) to conduct RNA-seq profiling in the roots of *G. barbadense* resistant (Pima-S6) and susceptible cultivars (Pima S-7 and Pima 3-79) and determined the genes that are differentially expressed in Pima-S6 in response to FOV4 infection. RNA-seq data indicates that the transcriptional response of Pima-S6 differs from that of susceptible genotypes in magnitude (a smaller number of differentially expressed genes) and functional enrichment (Figure 2). GO functional analysis of the genes activated in Pima-S6 in response to FOV4 infection revealed a differential activation of metabolic pathways that do not occur in susceptible genotypes Pima 3-79 and Pima S-7 (Figures 3A, B).

**FIGURE 5:**
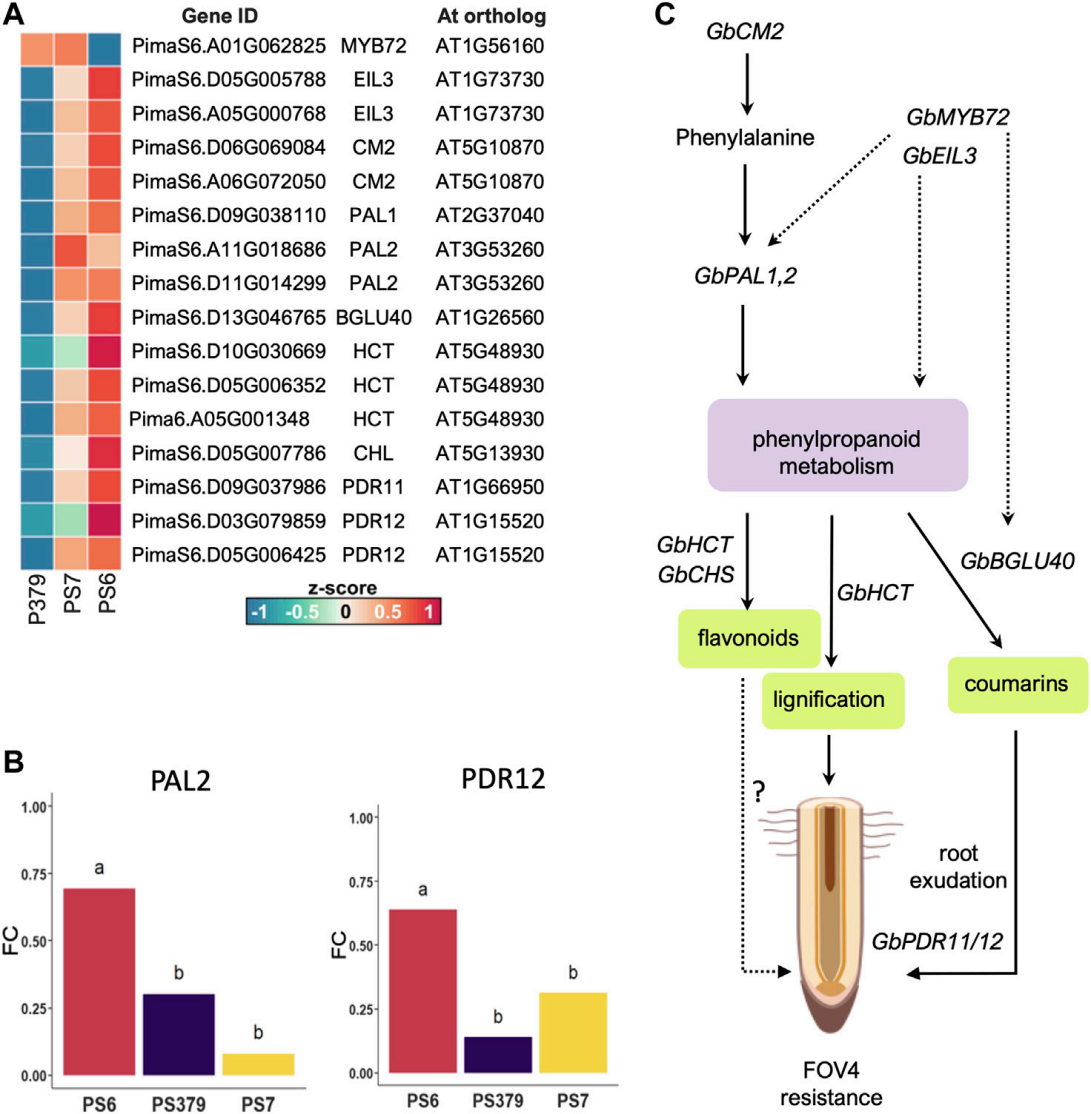
Phenylpropanoid metabolism and the possible genes and pathway that contribute to FOV4 resistance in Pima-S6. **(A)** Heatmap of relative expression (z-score) of candidate genes that contribute to the enhanced phenylpropanoid metabolism observed in Pima-S6 (PS6) and their Arabidopsis ortholog, compared to Pima S-7 (PS7) and Pima 3-79 (P379). **(B)** Relative quantification by RT-qPCR of mRNA expression levels (fold change, FC) of PAL2 (left) and PDR12 (right) compared to the expression level of UBQ14. Different letters indicate statistically significant differences according to Tukey’s HSD test (*p* < 0.05). **(C)** Putative gene regulatory pathway that controls the enhancement of phenylpropanoid metabolism and is upregulated in response to FOV4 in PS6. Chorismate mutase (encoded by *GbCM2*) catalyzes phenylalanine synthesis, which boosts the phenylpropanoid pathway via phenylammonia lyase activity (encoded by *GbPAL1*). Three possible pathways contribute to FOV4 resistance: coumarin exudation, lignification of the root, and flavonoid synthesis. We observed enhanced levels of the coumarin esculetin, which suggests a basal synthesis of coumarins is enhanced in PS6; the exudation of these types of compounds could be enhanced via *GbBGLU40* and root ABC-type transporter-encoding genes *GbPDR11/12*. The same root exudation mechanisms could be operating for flavonoid compounds whose synthesis could be heightened by enhanced hydroxycinnamoyltransferase activity (encoded by *GbHCT* genes) and chalcone synthase activity (encoded by *GbCHS* genes). Increased lignification of the root of PS6, which we have observed in a previous study (Mendu et al., 2022), could be caused by the enhanced activity of the encoded products of *GbCM2*, *GbPAL1*, and *GbHCT*, which results in the secondary cell-wall thickening and provides a physical barrier of defense against the FOV4 threat. Parts of this figure were generated with BioRender.

Enhancement of secondary metabolism in response to FOV4 treatment, particularly of pathways related to the production of phenylpropanoids, is a key difference that sets the transcriptional response of Pima-S6 apart from that of Pima S-7 and Pima 3-79. Importantly, the activation of genes related to signaling and metabolism of phenylpropanoids was detected in non-infected roots of Pima-S6, suggesting these cotton plants are ready to respond to FOV4 infection (Supplementary Figure S2). Among the differentially enriched GO categories activated in the Pima-S6 transcriptional response to FOV4 treatment, we identified categories related to phenylpropanoid metabolism, including “ammonia-lyase activity” and “chorismate metabolic process”. The finding of these categories is interesting because an enhanced phenylpropanoid metabolism has been previously related to disease resistance in tobacco, rice, and barley (Shadle et al., 2003; Hu et al., 2009; Tonnessen et al., 2015; Jan et al., 2021). Identification of ortholog Arabidopsis genes to genes differentially expressed in FOV4-infected cotton plants revealed that genes orthologous to AtPAL1 and AtPAL2 are overexpressed in Pima-S6 with respect to the susceptible cotton cultivars (Supplementary Dataset S1; Figure 5A). PAL1,2 have been thoroughly related to plant defense responses (Huang et al., 2010; Piofczyk et al., 2015; Li et al., 2022). Our metabolomic analysis further corroborated transcriptomic findings as we found significant enrichment of the phenylpropanoid pathway in the roots of Pima-S6 compared to Pima S-7. We also identified enhanced transcript levels of *AtCHS1* orthologs in Pima-S6 (Supplementary Dataset S1; Figure 5A), suggesting a possible role of flavonoids in the FOV4 resistance mechanism in this cotton cultivar. Supporting this notion, we also found significant enrichment of flavonoids in the root metabolome of Pima-S6 compared to Pima S-7 (Figure 4D). Coding for the enzyme that catalyzes the first step in the synthesis of flavonoids, the expression of chalcone synthase genes is activated by plants in response to multiple stress conditions, including fungal infection (Dao et al., 2011). A previous study demonstrated that *AtCHS1* protects against the pathogenic fungi *Verticillium dahliae* in Arabidopsis and that knockdown of orthologs of this gene in cotton (*G. hirsutum*) results in enhanced susceptibility to *V. dahliae* infection (Lei et al., 2018).

To test the idea that Pima-S6 produces and exudates more phenolic compounds than Pima 3-79 and Pima S-7, we decided to analyze the expression of four genes related to the phenylpropanoid metabolism and root exudation of secondary metabolites, PAL1, PAL2, CM, and PDR12 (Pang et al., 2022), by RT-qPCR. We found clear enhanced expression of PAL2 and PDR12 in non-infected Pima-S6 roots (Figure 5B), whereas no significant difference in CM and PAL 1 expression levels between the three cotton varieties was found (Supplementary Figure S4). This suggests PAL2 and PDR12 might play important roles in the FOV4 resistance phenotype, as they are upregulated upon FOV4 infection. In Arabidopsis, PDR12 is upregulated in response to fungal infections (Abuqamar et al., 2013) and its mutation leads to pathogen susceptibility (Pang et al., 2022), demonstrating that PDR12 is an important component of the plant defense response to fungi. The fact that PDR12 is important in various plant species to defend against fungi suggests that it is more important than previously thought. Thus, it is an interesting candidate gene for breeding efforts that can be coupled with PAL genes.

Phenylpropanoid metabolism is closely linked to lignin deposition in the root; the synthesis of the main lignin subunits, monolignols, starts with the phenyl ammonia lyase reaction and continues with subsequent biochemical reactions that give rise to monolignols, which are then polymerized during secondary cell wall formation (Fraser and Chapple, 2011). It was recently reported that higher levels of lignin are associated with a higher resistance of Pima-S6 to FOV4 (Mendu et al., 2022). The significant enrichment of the phenylpropanoid pathway found in our transcriptomic (Figures 3B, C) and metabolomic (Figure 4D) analyses supports increased lignification in Pima-S6 as one of the mechanisms underlying FOV4-resistance in this cultivar. Enhanced expression of Pima-S6 orthologs of *AtHCT* (Supplementary Dataset S1; Figure 5A)*,* whose expression is positively correlated with lignin deposition (Liu et al., 2018), provides candidate genes to explain the enhanced lignification in Pima-S6. Interestingly, we also found significant enrichment of cutin, suberin, and wax biosynthesis in Pima-S6 compared to Pima 3-79 (Figure 4D). Phenylpropanoid metabolism has recently been shown to be essential for tissue-specific suberin deposition in the root endodermis (Andersen et al., 2021). Both suberin and lignin contribute to forming root cell barriers that, among other functions, protect the plant from pathogen invasion. Concomitantly, lignin deposition has been recently shown to be part of a disease-resistance mechanism against *Verticillium dahlia* and *Fusarium oxysporum* in Upland cotton (Li et al., 2021).

Identified for the first time in the plant species formerly known as *Coumarona odorata,* coumarins is a family of secondary metabolites derived from the phenylpropanoid pathway (Stringlis et al., 2019). Coumarins consist of a benzene ring linked to a pyrone ring and have been linked with Fe uptake and, recently, with regulating plant-microbe interactions (Clemens and Weber, 2016; Stringlis et al., 2019). The coumarin esculetin was reported to present antifungal activity, i.e., by inhibiting *Phytophthora capsici* growth *in planta* and *in vitro* (Wang et al., 2021)55. Interestingly, we found enhanced levels of esculetin in the metabolome of Pima-S6 roots (Figure 4F; Supplementary Figure S3), which suggests this molecule might be involved in the FOV4-resistance response in this cotton variety. A recent report highlighted the role of the transcription factor *AtMYB72* in activating coumarin exudation to modulate plant-microbe interactions and protect Arabidopsis from *F. oxysporum* infection (Stringlis et al., 2018). The activation of the expression of the *BETA-GLUCOSIDASE 42 (AtBGLU42)* gene by *AtMYB72* promotes the de-glycosylation of scopolin into scopoletin, which facilitates its exudation into the rhizosphere. Scopoletin was reported to inhibit *F. oxysporum* growth *in vitro* (Stringlis et al., 2018). We found higher transcript levels of two of *AtBGLU40* in Pima-S6 (Supplementary Dataset S1; Figure 5A), which belongs to the same family of beta-glucosidases with coumarin-hydrolyzing activity as *AtBGLU42* (Dong et al., 2019) and whose enzymatic activity is enhanced upon *F. oxysporum* infection (Husaini et al., 2018). We also found that Pima-S6 presents an enhanced expression of *AtPDR11/12* orthologs in response to FOV4 treatment (Supplementary Dataset S1; Figure 5A), which encode ABC-type transporters that facilitate the exudation of secondary metabolites to the rhizosphere (Ziegler et al., 2017; He et al., 2019).

Following the *AtMYB72-GLU42* model (Stringlis et al., 2018), we looked for *GbMYB72* orthologs and found only one hit in our assembly of the Pima-S6 genome (Supplementary Dataset S1; Figure 5A), but it was not significantly upregulated by FOV4 treatment in any of the tested genotypes. However, it has been reported that over-expression of *MYB72* does not enhance disease resistance (Van Der Ent et al., 2008), suggesting that this transcription factor is most likely subject to a post-translational regulation mechanism. Furthermore, it has been demonstrated that *AtMYB72* interacts with the *ETHYLENE INSENSITIVE LIKE 3 (EIL3)*, suggesting a role for this transcriptional activator in regulating *AtMYB72* activity (Van Der Ent et al., 2008). Nonetheless, the nature of the relationship between these two transcriptional activators has yet to be elucidated. Interestingly, we found ethylene signaling as one of the phytohormone GO categories enriched in Pima-S6 (Figure 4C). Moreover, we found two *GbEIL3* orthologs significantly upregulated in Pima-S6 in response to FOV4 treatment compared to the susceptible cotton genotypes tested (Supplementary Dataset S1; Figure 5A).

Figure 5C illustrates the possible pathways and candidate genes contributing to FOV4 resistance in Pima-S6 cultivar. We provide metabolomic and transcriptomic evidence that indicates a central role for phenylpropanoid metabolism in FOV4 resistance. The enhanced expression of *GbCM2* and *GbPAL1,2* genes in FOV4-infected Pima-S6 plants leads to an increased phenylpropanoid metabolism. In Pima-S6, lignification might be increased by the FOV-4-induced phenylpropanoid metabolism and the increased expression of *GbHCT* genes*,* which contributes to forming a physical barrier for FOV4 infection. Transcriptional activation of *GbCM2* and *GbPAL1* can take place by transcription factors like *GbMYB72 and GbEIL3.* These transcription factors might also interact to activate the expression of *GbPAL1* and *GbBGLU40* to facilitate coumarin de-glycosylation and further exudation, which is toxic for *F. oxysporum* (Stringlis et al., 2018). Flavonoid synthesis, enhanced through the upregulation of phenylpropanoid metabolism and *GbHCT and GhCHS* genes, might also contribute to the FOV4 resistance. Lastly, enhanced expression of ABC-type transporters through *GbPDR* genes might ultimately contribute to the exudation of flavonoids and coumarins, which are toxic for FOV4. Previous QTL studies have highlighted *G. barbadense* as a source of FOV4 resistance (Ulloa et al., 2013; Ulloa et al., 2016a; Wang et al., 2018). The genes presented in Figure 5A, which include GbCM2 paralogs (PimaS6.A06G072050, PimaS6.D06G069084) and GbPDR12 (PimaS6.D03G079859) are interesting prospects of research for FOV4-disease resistance; their sequences and exact chromosomic locations are publicly available and can be found in the Pima-S6 genome assembly (Chávez Montes et al., 2023). Overall, our study indicates that genes linked to enhanced phenylpropanoid metabolism are attractive research targets for transferring FOV4 resistance to other cotton cultivars of economic relevance.

## Data Availability

The datasets presented in this study can be found in online repositories. The names of the repository/repositories and accession number(s) can be found in the article/Supplementary Material.
