# Supplementary material for: Enhanced phenylpropanoid metabolism underlies resistance to Fusarium oxysporum f. sp. vasinfectum race 4 infection in the cotton cultivar Pima-S6 (Gossypium barbadense L.)
Source: Front Genet. 2024 Jan 8;14:1271200. doi: 10.3389/fgene.2023.1271200 (PMC10800685; doi:10.3389/fgene.2023.1271200)
Supplement: Supplementary file 1 [file Table1.pdf]

Supplementary Material

Enhanced phenylpropanoid metabolism underlies resistance to *Fusarium oxysporum* f. sp. *vasinfectum* race 4 infection in the cotton cultivar Pima-S6 (*Gossypium barbadense* L.)

Jonathan Odilón Ojeda-Rivera[1], Mauricio Ulloa[2,*], Francisco G. Perez-Zavala[1], Héctor-Rogelio Nájera-Gonzalez[1], Philip A. Roberts[3], Lenin Yong-Villalobos[1], Himanshu Yadav[1], Ricardo A. Chávez Montes[1], Luis Herrera-Estrella[1,4], Damar Lopez-Arredondo[1,*]

[1]Institute of Genomics for Crop Abiotic Stress Tolerance, Plant and Soil Science Department, Texas Tech University, Lubbock, TX, USA.
[2] Plant Stress and Germplasm Development Research, U.S. Department of Agriculture-Agricultural Research Service, Plains Area, Cropping Systems Research Laboratory, Lubbock, TX, USA.
[3]Department of Nematology, University of California, Riverside, CA, USA.
[4]Unidad de Genomica Avanzada/Langebio, Centro de Investigacion y de Estudios Avanzados, Irapuato, Guanajuato, Mexico.

*** Correspondence:**
Damar Lopez-Arredondo
Damar.Lopez-Arredondo@ttu.edu
Mauricio Ulloa
mauricio.ulloa@usda.gov

Keywords: Pima cotton, *F. oxysporum* race 4-resistance, phenylpropanoid metabolism, Fusarium wilt, RNA-seq analysis

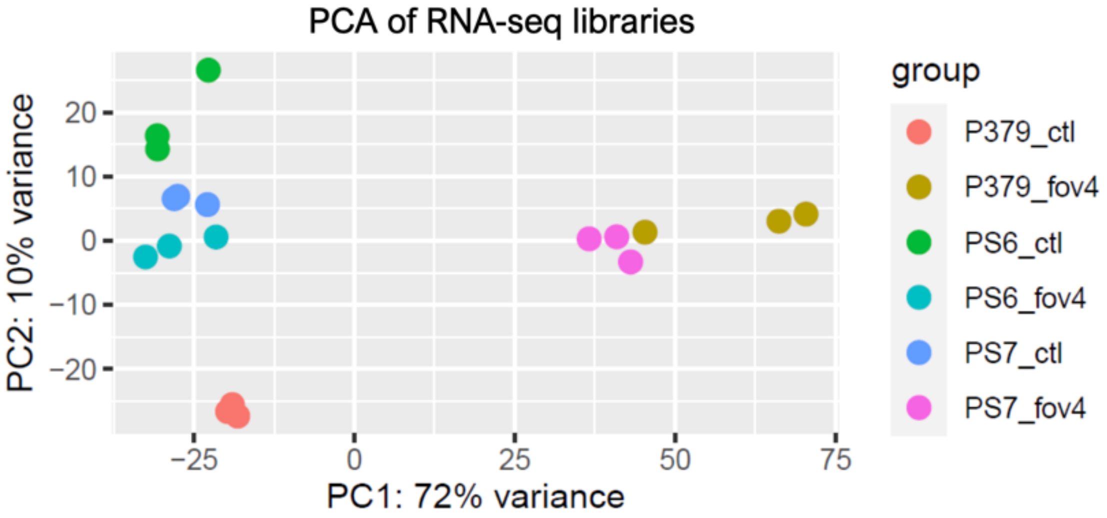

Supplementary Figure S1. Principal Component Analysis (PCA) of the RNA-seq libraries generated in this study.

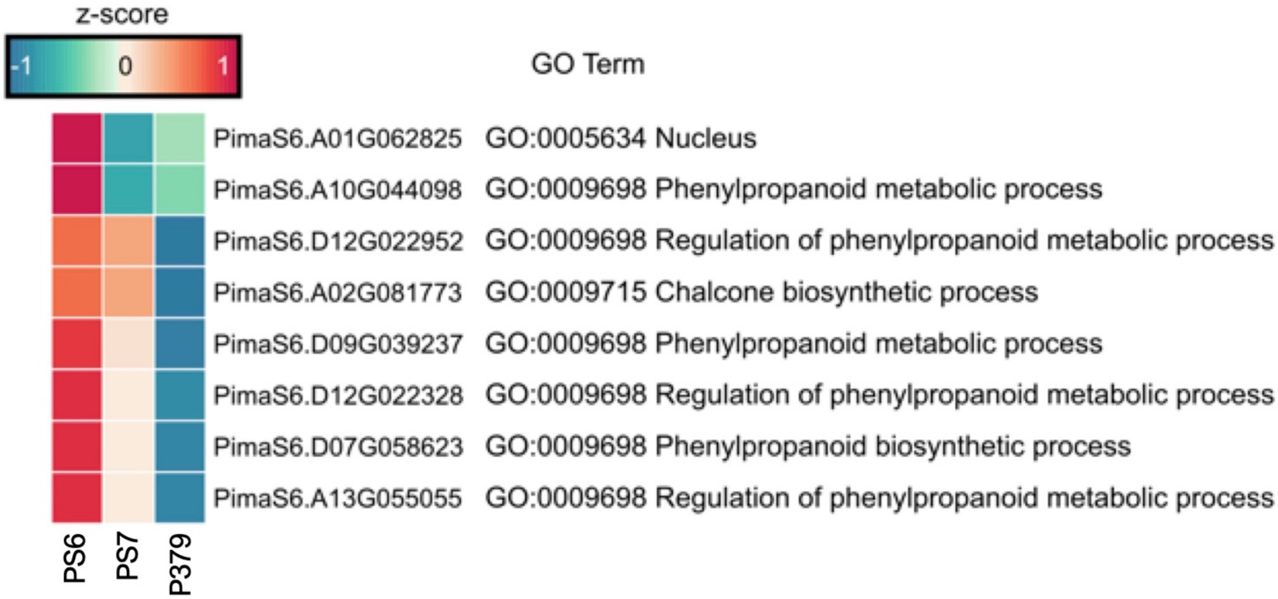

Supplementary Figure S2. Heatmap of relative expression (z-score) of genes belonging to Gene Ontology categories related to regulation and metabolism of phenylpropanoids upregulated in Pima-S6 (PS6) compared to Pima S-7 (PS7) and Pima 3-79 (P379), in the non-infected control samples.

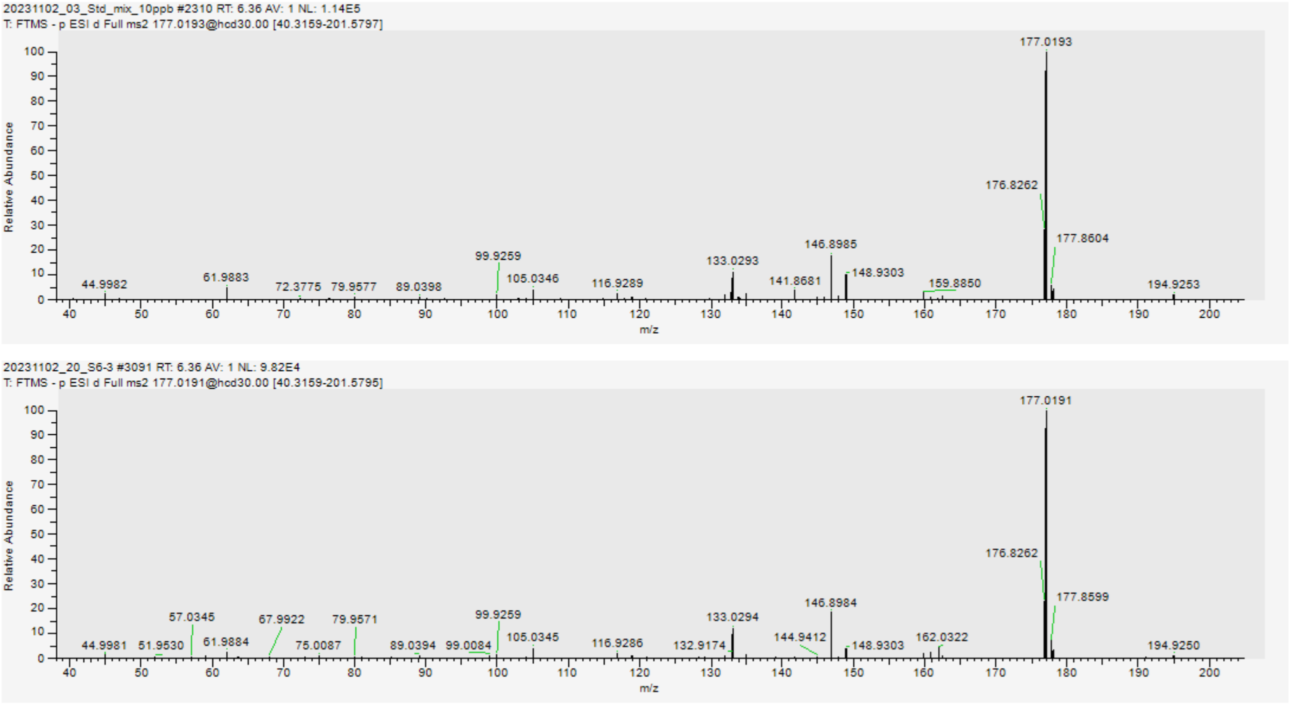

Supplementary Figure S3. Esculetin $[M-H]^-$=177.0193 $[C_9H_5O_4]^-$ Full MS2 spectrum from an analytical standard (upper panel) and the cotton root sample of Pima-S6 cotton (lower panel).

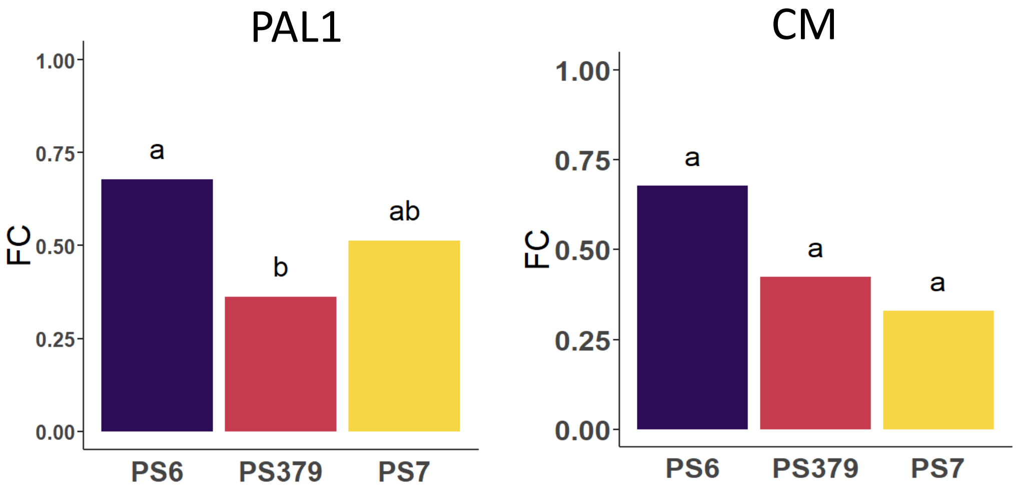

Supplementary Figure S4. Relative quantification by RT-qPCR of mRNA levels (fold change, FC) of PHENYLALANINE AMMONIA LYASE (PAL1, left) and CHALCONE SYNTHASE (CM, right), compared to the expression level of the internal control UBQ14. Different letters indicate statistically significant differences according to Tukey's HSD test ($p < 0.05$).

Supplementary Table S1. Alignment statistics and information about the 18 RNA-seq libraries generated and analyzed in this study. All libraries were sequenced using Illumina technology. Pima libraries were aligned to the assembly of the Pima-S6 *G. barbadense* genome (Chávez Montes et al., 2023).

Genotype	Treatment	Replicate	Raw Reads	Aligned Reads	% of Alignment	Library Type
Pima 3-79	control	1	4217918	3521586	83.5	Paired-end
Pima 3-79	control	2	4484855	3766385	84.0	Paired-end
Pima 3-79	control	3	12685829	9842536	77.6	Paired-end
Pima 3-79	FOV4	1	4540990	3490687	76.9	Paired-end
Pima 3-79	FOV4	2	3881310	3065877	79.0	Paired-end
Pima 3-79	FOV4	3	4166535	2937874	70.5	Paired-end
Pima-S6	control	1	4206901	3456981	82.2	Paired-end
Pima-S6	control	2	4264099	3578609	74.6	Paired-end
Pima-S6	control	3	13306590	9466152	71.1	Paired-end
Pima-S6	FOV4	1	4600488	3810780	82.8	Paired-end
Pima-S6	FOV4	2	4506533	3729056	82.7	Paired-end
Pima-S6	FOV4	3	9363353	6879433	73.5	Paired-end
Pima-S7	control	1	4510900	3758257	83.3	Paired-end
Pima-S7	control	2	4226092	3502960	82.9	Paired-end
Pima-S7	control	3	11037955	8689885	78.7	Paired-end
Pima-S7	FOV4	1	4117433	3285851	79.8	Paired-end
Pima-S7	FOV4	2	4072252	3294645	80.9	Paired-end
Pima-S7	FOV4	3	10450239	7771473	74.4	Paired-end
